# Supplementary material for: Taxonomic similarity does not predict necessary sample size for ex situ conservation: a comparison among five genera
Source: Proc Biol Sci. 2020 Apr 29;287(1926):20200102. doi: 10.1098/rspb.2020.0102 (PMC7282903; doi:10.1098/rspb.2020.0102)
Supplement: Supplementary Methods and Results [file rspb20200102supp1.docx]

**SUPPLEMENTAL MATERIAL FOR PAPER “**The optimal size of an ex situ conservation population: a comparison among 11 taxa in 5 genera”

**Table of contents**

1. Introduction to study species (expanded Table 1)
2. Acknowledgements of Gardens that contributed ex situ tissue
3. Details of field sampling and laboratory genotyping for each species
   1. Quercus
   2. Magnolia
   3. Hibiscus
   4. Pseudophoenix
   5. Zamia
4. Additional Results
   1. Genetic diversity statistics for all wild populations for each species
   2. Allele capture curves and minimum sampling for 70% sufficiency
   3. P values for statistical tests
      1. F_ST_
      2. Allele frequency bins summary for each species

Introduction to Study Species

Expanded Table 1 with additional comments, and threat status.

| **Species** | **In situ Range** | **Additional comments** | **IUCN Red List / NatureServe rank if U.S. native** |
| --- | --- | --- | --- |
| *Hibiscus waimeae* subsp*. hannerae* | several valleys on Kauaʻi, HI | Decline/fragmentation, invasive species, possible loss of native pollinators | CR / G2T1 |
| *Hibiscus waimeae* subsp*. waimeae* | one canyon and few valleys on Kauaʻi, HI | Some populations show signs of recruitment | NT / G2T2 |
| *Magnolia pyramidata* | ~10 counties in FL | Significant loss of mature individuals due to hurricane damage | LC/ G4 |
| *Magnolia ashei* | highly scattered in GA, AL, FL, MS, LA, TX | Decline/ fragmented | VU** / G2 |
| *Pseudophoenix ekmanii* | one park in the Dominican Republic | Decline from poaching | CR |
| *Pseudophoenix sargentii* | Yucatan, Belize, several Caribbean islands | Decline from poaching | G4 |
| *Quercus boyntonii* | few counties in AL | Land use change | CR / G1 |
| *Quercus georgiana* | few counties in AL, GA, SC, NC | Land use change | EN/ G3 |
| *Quercus oglethorpensis* | ~10 counties in LA, MS, AL, GA, SC | Land use change | EN / G3 |
| *Zamia decumbens* | Toledo District, Belize | Decline from poaching | CR |
| *Zamia lucayana* | Narrow beach on Long Island, Bahamas | Stable | EN |

** Assessed as *Magnolia macrophylla* var*. ashei*.

**Figure showing species distributed on the seed plant tree of life**

Note that not every species is shown due to space.


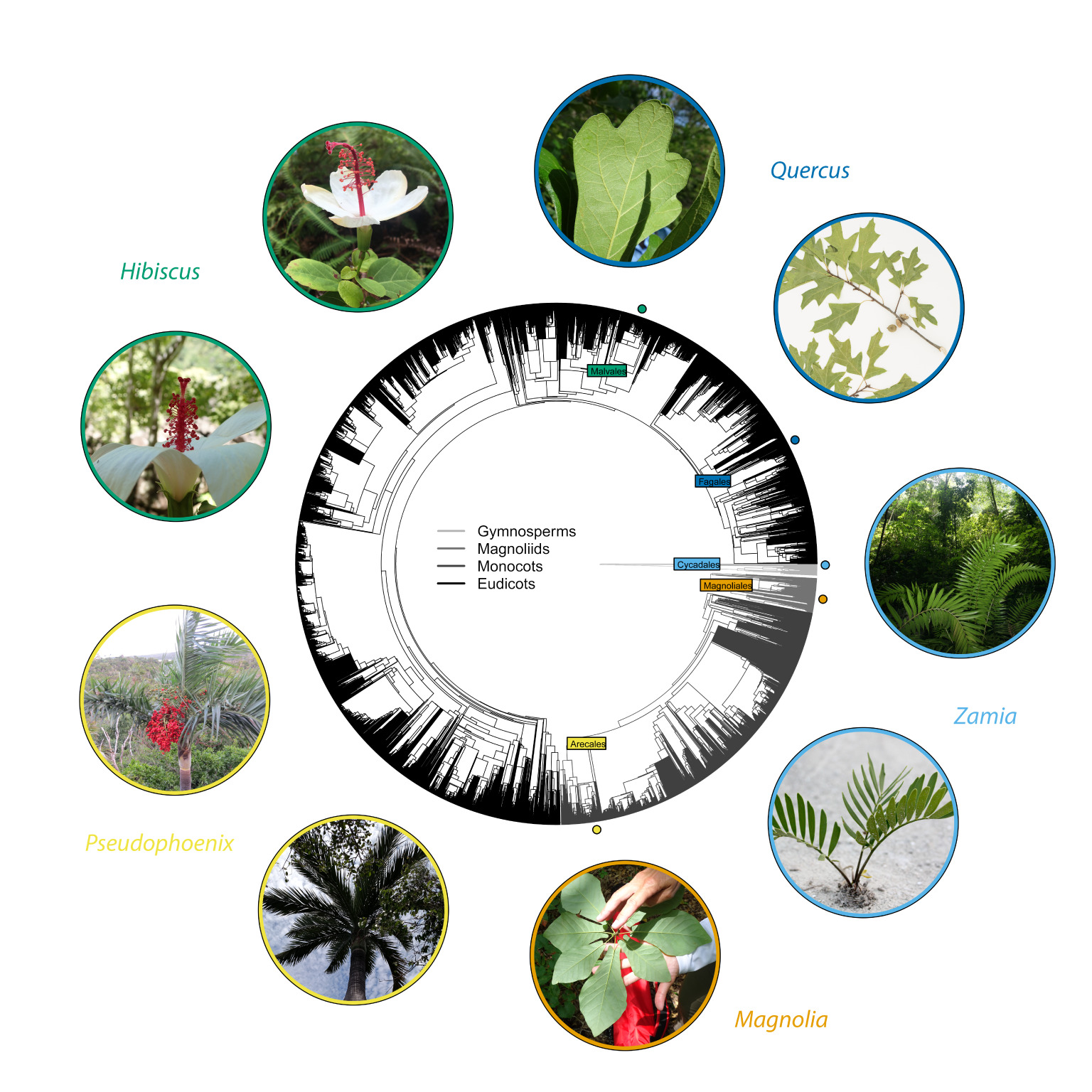


**Acknowledgement of garden contributions**

We thank the following gardens for sending tissue of plants that they are conserving

| Aiken Citywide Arboretum |
| --- |
| Arboetum des Grandes Bruyères |
| Arboretum de Pezanin |
| Arboretum de Ripaille |
| Arboretum des Pouyouleix |
| Arboretum Wespelaar |
| Arnold Arboretum, Boston, MA—from plant in Gladwyne, PA |
| Atlanta Botanical Garden |
| Australian BG. Mount Annan |
| Barnes Foundation |
| Bartlett Tree Research Laboratories Arboretum |
| Bishop Museum |
| Botanic Garden Meise |
| Botanische Gärten der Universität Bonn |
| Brookside Gardens, Wheaton, MD—from commercial nursery in Tallahassee, FL |
| Charles R. Keith Arboretum |
| Chicago Botanic Garden |
| Dawes Arboretum |
| Donald E Davis Arboretum |
| ENGREF - Arboretum National des Barres |
| Gainesway Farm |
| Garret Park, MD |
| Hawaii Tropical BG |
| Holden Arboretum |
| Hoyt Arboretum |
| Jardin Botanico Nacional Rafael Moscoso (DR) |
| JC Raulston Arboretum |
| Lady Bird Johnson Wildflower Center |
| Longwood Gardens |
| Missouri Botanical Garden |
| Moalepe |
| Montgomery Botanical Center |
| Moore Farms Botanic Garden |
| Mt. Cuba Center |
| National Arboretum/GRIN |
| National Tropical BG |
| Newberry St, Aiken, SC |
| North Carolina Arboretum |
| Oglethorpe County Courthouse |
| Private Garden |
| Red’s Rhodies, Sherwood, OR |
| Royal Botanic Gardens (Kew) |
| Royal Botanic Gardens (Wakehurst) |
| Sarah P. Duke Gardens |
| Schoenike Arboretum at South Carolina Botanical Garden |
| South Carolina Botanic Garden, Clemson, SC |
| Starhill Forest Arboretum |
| State Arboretum of Virginia |
| Superior Trees, Lee, FL—collected in Leon Co., FL |
| Taltree Arboretum & Gardens |
| The Keith Arboretum |
| The Morris Arboretum |
| The Morton Arboretum |
| The Scott Arboretum |
| The State Botanical Garden of Georgia |
| Thompson Mills Forest |
| Thurmont, MD—Gordon Hagen |
| United States Botanic Garden |
| Univ. of Delaware Botanic Gardens, Newark, DE—from Woodlander’s Nursery |
| University of Washington Botanic Garden |
| Waimea Arboretum |
| Woodlander’s Nursery, Aiken, SC |

**Species sampling**


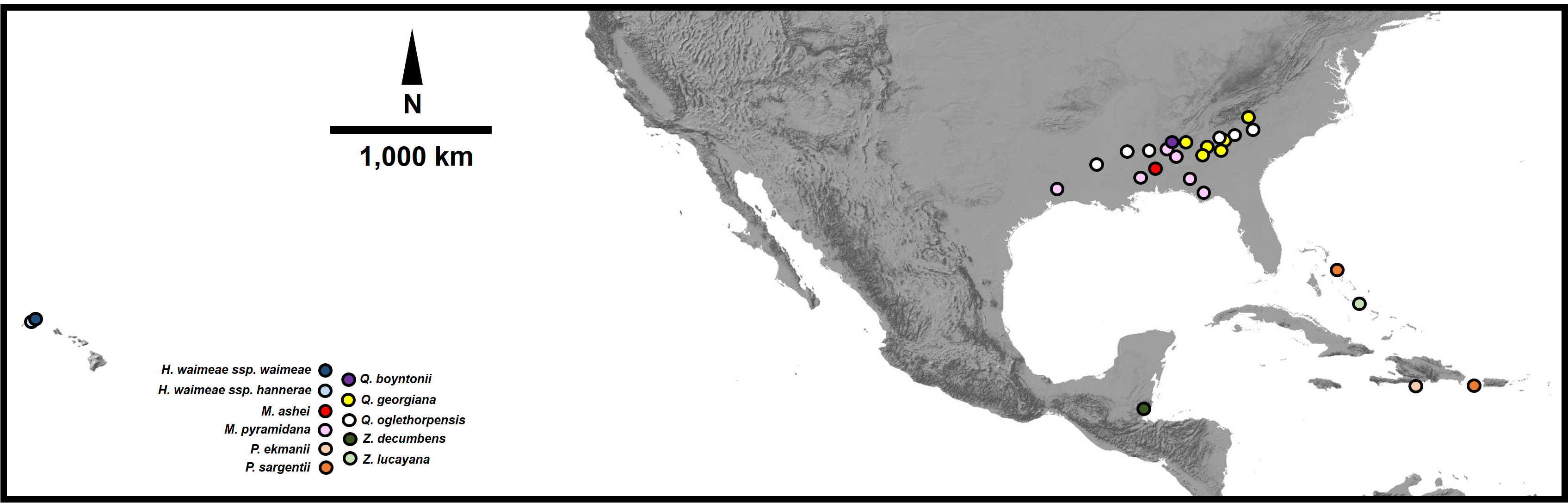


*Generic locations of field sampling are shown above, reflecting the species’ distributions. For those species not threatened by poaching we include lat/long under each species below*

***Quercus boyntonii***

*Quercus boyntonii* was sampled in situ from known wild populations in several natural preserves, private property, and suburban parks, and ex situ from all known botanic gardens and arboreta that have *Q. boyntonii* in their living collections. In situ, population sizes ranged from fewer than 10 to more than 100 trees. Occurrences are patchy, coinciding with suitable remnant habitat- sandstone outcrops, ridges, and slopes. Because of patchiness of habitat and occurrence, and wind pollination in oaks, it is challenging to delimit strict “populations”. We selected only trees that had typical *Q. boyntonii* leaf shape and size, although it is possible some hybrids could have been sampled because gene flow among oak species and interrogations among oak species are commonly observed.

We sampled, 246 in situ samples (227 included in final analysis) and 77 ex situ samples (all 77 included in the final analysis, QB Table 1). In situ samples were collected during May 2017, and ex situ samples were collected between April and September 2017. In situ, in most cases, we intentionally avoided possible clones (stems located fewer than 5 meters apart) as the species is known to occur in small clonal patches. 3 to 5 whole, non-damaged leaves were sampled, when possible. The samples were kept on ice in the field/during transport and then in a +4°C fridge, for up to one week. When the samples arrived at the laboratory, they were kept at -80°C until DNA extraction. All individuals were given a unique identification number, in situ individuals were additionally geolocated, and had basic metrics measured (e.g., DBG, general health, etc.). In situ population names, number of samples genotyped, and population centroids are given in QB Table 2.

QB Table 1: Sampled living collection tissues included in the study for *Q. boyntonii*.

| **Garden** | **State** | **Country** | **Accession No.** | **Accession origin** |
| --- | --- | --- | --- | --- |
| Atlanta Botanical Garden | GA | USA | ABG20170679 | Mountain Brook, AL |
| Bartlett Tree Research Laboratories Arboretum | NC | USA | 2002-036*A,2002-037*A | Horticultural |
| Chicago Botanic Garden | IL | USA | 1672-2015, 1672-2015, 1672-2015, 1672-2015, 1672-2015, 1972-2015, 1672-2015, 1677-2015, 1670-2015, 1670-2015, 1670-2015, 1670-2015, 1676-2015, 1676-2015, 1677-2015 | Wild, plant collecting trip |
| Gainesway Farm | KY | USA | N/A | National Arboretum (NA-77492-001) |
| Missouri Botanical Garden | MO | USA | 2014-1958-1, 2014-1985-2 | Wild, Alabama |
| Moore Farms Botanic Garden | SC | USA | 1545m15-1, 1545m15-2, 1545m15-3, 1553m15-1, 1553m15-2, 1553m15-3 | Wild, Alabama |
| Mt. Cuba Center | DE | USA | 20080437 | Wild, Alabama |
| National Arboretum/GRIN | DC | USA | NA77492 | Wild, Alabama |
| Starhill Forest Arboretum | IL | USA | Lot 3, Lot 1 | Wild, Gadsden AL |
| The Donald E. Davis Arboretum | AL | USA | 2016031, 2016032, 2015052, 2016030, 2011076, 2011077.1, 2012217.2, 2012217.3, 2012217.1, 2015054 | Wild, Alabama |
| The Keith Arboretum | NC | USA | KA3319, KA3319 | Unknown |
| The Morris Arboretum | PA | USA | 2009-067*A, 2009-067*B | Mt. Cuba Center |
| The Morton Arboretum | IL | USA | 639-2015, 639-2015, 639-2015, 639-2015, 639-2015, 639-2015, 386-2010-1, 639-2015, 634-2015, 634-2015, 634-2015, 635-2015, 635-2015, 635-2015, 635-2015, 633-2015, 633-2015, 633-2015, 633-2015, 633-2015, 633-2015, 633-2015, 636-2015, 636-2015, 632-2015, 632-2015, 635-2015 | Wild, Plant collecting trip |
| Lady Bird Johnson | TX | USA | QUBO_WC01A, QUBO_WC01B, QUBO_WC01C, QUBO_WC01D, QUBO_WC01E, QUBO_WC01F, QUBO_WC01G, QUBO_WC01H, QUBO_WC01I, QUBO_WC01J | Wild, near Gadsden, AL |

QB Table 2: In situ *Q. boyntonii* population information

| **Population Name (ID)** | **Number of samples Genotyped** | **Population State** | **Population County** |  |  |
| --- | --- | --- | --- | --- | --- |
| Worldsong (2) | 14 | Alabama | St. Clair | -86.4 | 33.6 |
| Wattsville (3) | 18 | Alabama | St. Clair | -86.3 | 33.7 |
| Moss Rock Preserve (4) | 22 | Alabama | Jefferson | -86.9 | 33.4 |
| Hinds Road (7) | 60 | Alabama | Etowah | -86.0 | 34.1 |
| Oak Mountain State Park (6, 8, 9) | 83 | Alabama | Shelby | -86.7 | 33.4 |
| Peavine Falls (10) | 30 | Alabama | Shelby | -86.8 | 33.3 |

***Quercus georgiana***

Sampling occurred across the known range of the species, which was determined through the use of herbarium records, collection data from botanic garden records, and USDA PLANTS Database (USDA 2012). Nine populations of *Q. georgiana* were sampled from sites in Georgia and Alabama (QG Figure 1, QG Table 1). Two sites (Camp Meeting Rock in Heard County, Georgia and Panola Mountain, on the boundary between Henry County and Rockdale County, Georgia) were visited, but no collections were made as *Q. georgiana* was infrequent and/or trees encountered appeared to be hybrids. All sampled populations were separated by at least 15 kilometers. Voucher specimens for each site were deposited at Longwood Gardens Herbarium (KEN) and University of Georgia Herbarium (GA). A total of 215 samples consisting of the nine populations of *Quercus georgiana* were sampled in Alabama and Georgia (Table 1, Figure 1).

Leaves were collected from wild populations in June 2011. At least 24 individual trees were randomly sampled from each site, and sampled plants were at least five meters apart. GPS coordinates were recorded for each plant. Fresh leaves were stored on ice until they were transferred to a -80°C freezer. For living collections, fresh leaf material was shipped to Chicago Botanic Garden from 17 botanical institutions in the United States, France, and Belgium in 2011 and 2015, totaling 34 individuals of *Q. georgiana* (QG Table 2). Leaf material was immediately transferred to a -80°C freezer upon arrival at the Chicago Botanic Garden.

QG Table 1: Sampled living collections of *Q. georgiana* included in study.

| **Garden** | **State** | **Country** | **Accession No.** | **Accession origin** |
| --- | --- | --- | --- | --- |
| Bartlett Tree Research Laboratories Arboretum | NC | USA | 2004-624, 2008-0001, 93-2058, 93-2059, 94-2122, 94-2123 | Horticultural |
| Charles R. Keith Arboretum | NC | USA | N/A | Horticultural |
| Donald E. Davis Arboretum | AL | USA | 2008156 | Unknown |
|  |  |  | 2009114A, 2009114B | Harris Co., GA |
| The Morton Arboretum | IL | USA | 49-2002*1, 49-2002*3, 49-2002*4, 40-2003*5 | Stone Mountain, GA |
| Sarah P. Duke Gardens | NC | USA | N/A | Gwinnett Co., GA |
| Schoenike Arboretum at South Carolina Botanical Garden | SC | USA | SCBG.13M3, SCBG.13N2 | Unknown |
| Starhill Forest Arboretum of Illinois College | IL | USA | 1994-030 | Stone Mountain, GA |
|  |  |  | 1994-029 | Pine Mountain, GA |
| State Arboretum of Virginia | VA | USA | 1532 9253.41 | Stone Mountain, GA |
| The State Botanical Garden  of Georgia | GA | USA | 98-0221.1, 98-0221.2, 98-0221.3 | wild collected,  unknown location |
| Taltree Arboretum & Gardens | IN | USA | 2007.036 | Stone Mountain, GA |
| Arboretum des Pouyouleix |  | FRANCE | 0594, 01030 | Stone Mountain, GA |
| Arboretum de Pezanin |  | FRANCE | PEZANN | Stone Mountain, GA |
| Arboretum de Ripaille |  | FRANCE | AdRTlB | Stone Mountain, GA |
| ENGREF - Arboretum National des Barres |  | FRANCE | 5237, 5263, 5242 | Stone Mountain, GA |
| Arboetum des Grandes Bruyères |  | FRANCE | 45450 | Stone Mountain, GA |
| Private Garden |  | FRANCE | TIGY | Stone Mountain, GA |
| Arboretum Wespelaar |  | BELGIUM | 12265 | Stone Mountain, GA |

QG Table 2: In situ *Q. georgiana* study populations.

| **Locality** | **Pop.**  **Code** | **County** | **State** | **Latitude °N** | **Longitude °W** |
| --- | --- | --- | --- | --- | --- |
| Davidson-Arabia Mountain Nature Preserve | AM | DeKalb | GA | 33.6 | -84.1 |
| Chattahoochee Bend State Park | CB | Coweta | GA | 33.4 | -85.0 |
| Concord Road | CR | Pike | GA | 33.2 | -84.5 |
| Dowdell's Knob, F.D. Roosevelt State Park | DK | Harris | GA | 32.8 | -84.8 |
| Eden | ED | St. Claire | AL | 33.6 | -86.4 |
| Moss Rock Preserve | MR | Jefferson | AL | 33.4 | -86.8 |
| Penton | PN | Chambers | AL | 33.0 | -85.5 |
| Stone Mountain | SM | DeKalb | GA | 33.8 | -84.2 |
| Walnut Grove | WG | Walton | GA | 33.8 | -83.8 |
| North Carolina | NC | Rutherford | NC | 35.5 | -81.9 |

***Quercus oglethorpensis***

*Quercus oglethorpensis* is a long-lived woody plant endemic to the southeastern United States that was first described in 1940 (Duncan 1940). Extant, and largely fragmented, wild populations are documented in South Carolina, Georgia, Alabama, Mississippi and Louisiana (Kartesz 2015). We identified study sites using herbarium records and occurrence data. We prioritized sites with the most up-to-date occurrence data that was gathered in July 2015 during a germplasm collection effort (Lobdell and Thompson 2015). We included additional sites not visited during the collection effort so that the greatest geographic distribution could be sampled. The sampled populations were separated by at least 9 kilometers and sites ranged from 0.65km^2^ to 25km^2^. Site ownership consisted of federal (n=3), state (n=3), private (n=2), and conservation easement (n=1). Site habitat was markedly different across the range. At sites with less than 30 accessible individuals, all trees were sampled (minimum n=3). In sites with more than 30 individuals at least 24 haphazardly located individuals were sampled, and we avoided sampling from adjacent plants. At least one voucher specimen was collected from each site. Vouchers were later deposited at the Nancy Poole Rich Herbarium (Chicago Botanic Garden). GPS coordinates, diameter-at-breast-height (DBH) or height for plants less than 1m tall were recorded for all sampled plants.

Botanic gardens growing *Q. oglethorpensis* were identified using the Botanic Gardens Conservation International PlantSearch database of living collections and the BG-BASE Multisite plant search. Additional gardens were identified by personal communication with botanic garden professionals. Gardens were then contacted to determine the number of *Q. oglethorpensis* accessions, the number of individuals assigned to each accession, and the provenance of each accession. Leaf tissue samples were either dried in silica gel and shipped to the Chicago Botanic Garden, and then stored at -28ºC, or leaf material was collected fresh and immediately stored at -28ºC for later DNA extraction.

For the analysis, individuals of *Q. oglethorpensis* held ex situ were divided into two categories in order to assess the genetic diversity of the ex situ population before and after gap analysis sampling. The intention of the gap analysis sampling effort was to increase the genetic capture of the ex situ metapopulation. The collecting effort was led by the Morton Arboretum (Lisle, IL), and was sponsored by an American Public Garden Association and U.S. Forest Service Tree Gene Conservation Partnership grant in 2015. The targeted populations were in Mississippi, Alabama, Georgia, and South Carolina, because a qualitative assessment of current living collections showed no representation from Mississippi and limited representation from Alabama, Georgia, and South Carolina. The sampling resulted in several accessions from Alabama, but limited number of accessions from Mississippi and South Carolina. The populations in Louisiana were visited after this study was conducted, and a limited number of seeds are currently under stratification at Morton Arboretum (Lobdell and Thompson 2017).

QO Table 1: Number of accessions and individuals of *Quercus oglethorpensis* held in ex situ living collections. Note that three gardens do not report accession level records, so for the two gardens reporting more than one individual we do not know if the samples represent one or more accessions (i.e. one or more maternal source).

| **Ex situ site** | **Country** | **Number of accessions** | **Number of individuals** |
| --- | --- | --- | --- |
| Aiken Citywide Arboretum | United States | - | 2 |
| Arboretum Wespelaar | Belgium | 1 | 1 |
| Bartlett Tree Research Laboratories Arboretum | United States | 2 | 2 |
| Botanic Garden Meise | Belgium | 1 | 1 |
| Botanische Gärten der Universität Bonn | Germany | 1 | 1 |
| Chicago Botanic Garden | United States | 2 | 5 |
| Dawes Arboretum | United States | 3 | 3 |
| JC Raulston Arboretum | United States | 1 | 1 |
| Morton Arboretum^B^ | United States | 13 | 109 |
| Royal Botanic Gardens (Wakehurst) | England | 1 | 1 |
| Royal Botanic Gardens (Kew) | England | 1 | 1 |
| Sarah P. Duke Gardens | United States | 1 | 1 |
| Starhill Forest Arboretum | United States | 3 | 3 |
| United States Botanic Garden | United States | 1 | 1 |
| Thompson Mills Forest | United States | - | 12 |
| Oglethorpe County Courthouse | United States | - | 1 |
| **Total excluding gap analysis (i.e. Morton Arboretum)** | | 18 | 36 |
| **Total including gap analysis (i.e. Morton Arboretum)** | | 31 | 145 |

QO Table 2: in situ populations for *Quercus oglethorpensis*

| Site | County | State | N | Longitude | Latitude |
| --- | --- | --- | --- | --- | --- |
| Copenhagen Hills Preserve (LA-COP) | Caldwell | LA | 26 | -92.0 | 32.0 |
| RMS Timber (LA-RMS) | Caldwell | LA | 14 | -92.0 | 31.9 |
| Bienville National Forest (MS-BIE) | Scott | MS | 33 | -89.5 | 32.3 |
| County Road 14 (AL-CAT) | Marengo | AL | 27 | -87.5 | 32.2 |
| Monticello Glades (GA-MOT) | Jasper | GA | 31 | -83.7 | 33.3 |
| Buffalo Mill Road (GA-BUF) | Oglethorpe | GA | 29 | -83.0 | 33.9 |
| Goosepond Road (GA-GOS) | Oglethorpe | GA | 3 |  |  |
| Sumter National Forest (SC-SUM) | McCormick | SC | 27 | -82.2 | 33.9 |

Sites are listed from west to east with Copenhagen Hills Preserve (COP) being the westernmost site and Sumter National Forest (SUM) the easternmost site.

***Magnolia ashei***

*Magnolia ashei* was collected as described in Von Kohn et al. (2018).

***Magnolia pyramidata***

*Magnolia pyramidata* was sampled in situ from known wild populations in several natural preserves, private properties, and suburban parks and ex situ from known botanic gardens and arboreta that have *M. pyramidata* in their living collections. In situ, population sizes ranged from fewer than 10 to around 1,000 trees. Occurrences are patchy, coinciding with suitable remnant habitat of dense, rich wooded bluffs and ravine uplands.

We sampled 144 in situ samples and 41 ex situ samples (MP Table 1). In situ samples were collected during July 2017, and ex situ samples were collected between April and September 2017. In situ, individuals were sampled at an interval of 5 meters apart. 3 to 5 whole, non-damaged leaves were sampled, when possible. The samples were kept on ice in the field/during transport and then in a +4°C fridge, for up to one week. When the samples arrived at the laboratory, they were kept at -80°C. All individuals were given a unique identification number, in situ individuals were additionally geolocated, and had basic metrics measured (e.g., DBG, general health, etc.). In situ population names, number of samples genotyped, and population centroids are given in MP Table 2.

MP Table 1: Sampled living collection tissues included in the study for *M. pyramidata*.

| **Garden** | **State** | **Country** | **Accession No.** | **Accession origin** |
| --- | --- | --- | --- | --- |
| Atlanta Botanical Garden | GA | USA | ABG20120984, ABG20130989, ABG20120990 | Green Nurseries |
| National Arboretum/GRIN | DC | USA | NA62000 | Cultivated |
| National Arboretum/GRIN | DC | USA | NA80783 | Wild, Florida |
| National Arboretum/GRIN | DC | USA | NA80788 | Wild, Texas |
| Mt. Cuba Center | DE | USA | 2010392 | Wild, Alabama |
| Barnes Foundation | PA | USA | 1971/42 | Tom Dodd Nurseries |
| Chicago Botanic Garden | IL | USA | 2305-2016 | The Morton Arboretum |
| Chicago Botanic Garden | IL | USA | 1831-2016 | Stephen F. Austin University |
| Dawes Arboretum | FL | USA | D2012-0463.003, D2012-0464.001 | Wild, Florida |
| Holden Arboretum | OH | USA | 95-49 | Wild, Mississippi |
| The Morris Arboretum | PA | USA | 2001-224*A, 2001-225*B, 2001-226*B, 2001-223*I | Cultivated, Texas |
| University of Washington Botanic Garden | WA | USA | 442-61-A | Lufer Landscape (Salem, OR) |
| The Scott Arboretum | PA | USA | 2011-017 | Woodlanders Nursery |
| The Scott Arboretum | PA | USA | 2014-213 | Mt. Cuba Center |
| Hoyt Arboretum | OR | USA | 1993-059 | Purchased, 1993 |
| The Donald E. Davis Arboretum | AL | USA | 2010027.1, 2010027.2 | Superior Trees |
| Arboretum Wespelaar | N/A | Belgium | 1002 | Wild, Texas |
| Bartlett Arboretum | CT | USA | N/A | Unknown |
| North Carolina Arboretum | NC | USA | 1994-210*A | Seed collected from Marion, NC in 1992 |
| The Morton Arboretum | IL | USA | 403-2016, 425-2016, 425-2016, 425-2016, 425-2016, 425-2016, 403-2016, 403-2016, 403-2016, 403-2016, 403-2016, 403-2016, 403-2016 | Wild collecting trip |
| The Morton Arboretum | IL | USA | 47-94-1 | P Seitner, gift |

| Population Name (ID) | Number of samples Genotyped | Population State | Population County | longitude | latitude |
| --- | --- | --- | --- | --- | --- |
| Talladega National Forest (1) | 20 | Alabama | Bibb | -87.5 | 33.0 |
| Holland Bridge Road (2) | 1 | Alabama | Crenshaw | -86.4 | 31.7 |
| Pitt Springs (3) | 9 | Florida | Bay | -85.6 | 30.4 |
| Cat creek (4) | 3 | Florida | Bay | -85.6 | 30.4 |
| Beaver Creek (5) | 7 | Florida | Liberty | -85.0 | 30.5 |
| Greensboro (6) | 9 | Florida | Gadsden | -84.8 | 30.6 |
| Chattahoochee (7) | 13 | Florida | Gadsden | -84.9 | 30.6 |
| Selman (8) | 5 | Florida | Liberty | -85.0 | 30.5 |
| Sweetwater Tract of ABRP (Apalachicola Bluffs and Ravines Preserve) (9) | 5 | Florida | Liberty | -85.0 | 30.5 |
| Torreya State Park (10) | 9 | Florida | Liberty | -85.0 | 30.6 |
| Camp Shelby (11) | 10 | Mississippi | Forrest | -89.2 | 31.2 |
| Right-of-way  (Jasper County, TX) (12) | 8 | Texas | Jasper | -94.0 | 30.8 |
| Campbell Global (13) | 13 | Texas | Newton | -93.8 | 30.9 |

MP Table 2: In situ population information

***Hibiscus waimeae* subsp. *hannerae* and *H. waimeae* subsp. *waimeae***

For both subspecies of *Hibiscus waimeae*, individuals from all known wild populations were sampled during multiple trips from November, 2015 through August, 2017. The populations of *Hibiscus waimeae* subsp. *hannerae* occur on private property (Lower and Upper Limahuli Preserves, National Tropical Botanical Garden,) and state land (Nā Pali Coast State Wilderness Park, State of Hawaiʻi Department of Land and Natural Resources, Division of State Parks). A population is considered as all individuals occurring within a single valley, with Lower and Upper Limahuli being considered as separate valleys. Populations ranged from six (Pōhakuao) to approximately 100 individuals (Upper Limahuli Valley). However, in the Upper Limahuli Valley, 74 individuals total were accessible (others occurred in areas too steep to access). Two of the four populations are only accessible by helicopter followed by rough terrain hiking, including some rope work (Pōhakuao and Upper Limahuli Valley).The populations of *Hibiscus waimeae* subsp. *waimeae* occur throughout Waimea Canyon to ocean-facing valleys in the west and south-west of Kaua‘i*.* All in situ samples of *H. waimeae* subsp. *waimeae* for this study were collected on state land (State of Hawaiʻi Department of Land and Natural Resources, Division of Forestry and Wildlife) at the Great Escarpment, Koaie Canyon, Kukui Trail and Mahanaloa Valley. Populations ranged from three (Great Escarpment) to 41 individuals (Koaie Canyon). Leaf samples were placed into a paper coin envelope and into a re-sealable zipper storage bag containing silica beads within two days of collecting and then shipped to the Chicago Botanic Garden.

Individuals from which in situ leaf material was collected for this study were flagged and labeled with a unique identifier on a metal tag, geolocated, and had basic data recorded (e.g., plant height, phenology, general plant health, habitat description, associated species). Population (valley) names, number of samples collected and genotyped, and GPS coordinates for general population location, are given in HW Table 1 for *H. waimeae* subsp. *hannerae* and HW Table 2 for *H. waimeae* subsp. *waimeae*. Ex situ samples of both subspecies were obtained from all known botanic gardens and arboreta that have both or either taxon in their collections and allowed us to collect or were willing to send (HW Table 3).

HW Table 1. Ex situ samples of *Hibiscus waimeae* subspecies.

| *Gardens* | *Hibiscus waimeae* (undetermined subspecies) | *Hibiscus waimeae* subsp. *hannerae* | *Hibiscus waimeae* subsp. *waimeae* |
| --- | --- | --- | --- |
| Aust. BG. Mount Annan |  | 1 | 1 |
| Bishop Museum |  | 1 | 6 |
| Hawaii Tropical BG | 1 |  |  |
| Longwood | 1 |  |  |
| Missouri BG | 1 |  |  |
| Moalepe |  |  | 1 |
| National Tropical BG |  | 38 | 3 |
| Waimea Arboretum |  | 6 | 11 |
| Total | 3 | 46 | 22 |

HW Table 2: *Hibiscus waimeae* subsp. *hannerae* in situ population information

| Population Name | Number of samples Genotyped | Latitude | Longitude |
| --- | --- | --- | --- |
| Hanakāpiʻai | 48 | 22.2 | -159.6 |
| Lower Limahuli | 31 | 22.2 | -159.6 |
| Pōhakuao | 6 | 22.2 | -159.6 |
| Upper Limahuli | 74 | 22.2 | -159.6 |

HW Table 3. *Hibiscus waimeae* subsp. *waimeae* in situ population information

| Population Name | Number of samples Genotyped |  |  |
| --- | --- | --- | --- |
| Great Escarpment | 3 | 22.1 | -159.6 |
| Koaie Canyon | 41 | 22.1 | -159.6 |
| Kukui Trail | 13 | 22.1 | -159.7 |
| Mahanaloa | 31 | 22.1 | -159.7 |

***Pseudophoenix ekmanii***

*Pseudophoenix ekmanii* (Cacheito Palm) was sampled from two in situ populations in Parque Nacional Jaragua, Dominican Republic, and from ex situ populations at two botanic gardens that allowed access. PE Table 1 below. presents samples located and used in population genetic analysis. BGCI PlantSearch (BGCI 2016) indicated that this species was held in 5 ex situ sites. Contact was established among three sites, and two of these gardens allowed access to collections: Jardin Botanico Rafael Moscoso (JBSD) and Montgomery Botanical Center (MBC). Extensive in situ samples (n = 171) were collected by sampling every palm along feasible, meandering transects totaling 4.5 km through the subpopulations, taking into account limitations of access based on terrain (dogtooth limestone terraces), vegetation (seasonally dry thornscrub with abundant *Comocladia*; Figure 1), and logistics; plants are accessible by foot only, with no surface water resupply. Sayers (2009) describes helicopter access – such conveyance precludes only the last few kilometers of automobile and foot travel and does not allow for georeferenced sample collection. Two green leaflets were collected from each plant and placed in labeled resealable plastic bags for transport away from the field sites. Upon return to lodging (2-8 h) samples were cut into approximately 1cm2 squares and stored with silica gel desiccant.

**PE Table 1.** *Pseudophoenix ekmanii* samples used in this study.

| **Group** | **Description** | **N Plants** |
| --- | --- | --- |
| In situ |  |  |
| Sabana | Wild | 83 |
| Trudille | Wild subpopulation | 88 |
| Ex situ |  |  |
| Cohort 1 | MBC legacy collection (pre-2010) | 31 |
| Cohort 2 | JBSD legacy collection (pre-2016) | **17** |
| Cohort 3 | JBSD-MBC collection (2017) | 41 |

***Pseudophoenix sargentii***

*Pseudophoenix sargentii* (Buccaneer Palm, Palma Pirata) was sampled from three in situ populations, one of which was on Eleuthera, Bahamas, and two of which were on Mona Island (Puerto Rico), and from ex situ collections Montgomery Botanical Center directly derived from those in situ populations. PS Table 1 presents samples located and used in population genetic analysis. On Eleuthera, the in situ population is robust, continuous and numerous, whereas on Mona Island the two populations are quite small and isolated, allowing for total sampling (Santiago-Valentin et al. 2012). Sites were accessed via DNRA aircraft and then on foot. Two green leaflets were collected from each plant and placed in labeled resealable plastic bags for transport away from the field sites. Upon return to lodging (4-8 h) samples were cut into approximately 1cm2 squares and stored with silica gel desiccant.

PS Table 1. *Pseudophoenix sargentii* samples used in this study.

| **Location** | **Group** | **Description** | **N plants** |
| --- | --- | --- | --- |
| In situ | Antenna (Mona) | Wild population | 10 |
| In situ | Carabinero-Uvero (Mona) | Wild population | 10 |
| In situ | Eleuthera (Bahamas) | Wild population | 103 |
| Ex situ | Antenna | MBC collection (2012) | 12 |
| Ex situ | Carabinero-Uvero | MBC collection (2012) | 6 |
| Ex situ | Cohort 3 | MBC Collection (2017) | 81 |

***Zamia decumbens***

*Zamia decumbens* (Sinkhole Cycad) is known from a limited area of the Maya Mountains in southern Belize and is currently considered critically endangered (IUCN 2013). At the time of its description (Calonje et al. 2009), the species was known from two main populations of 234 and 183 plants, restricted to two limestone sinkholes separated by 7 km, and a few scattered hilltop populations of no more than 12 plants each. The remote, isolated locations preclude any potential introgression of other *Zamia* spp. from horticulture or in situ plants. This study focused on the two sinkhole populations and compares these wild plants to cultivated plants in MBC ex situ collections derived from these populations. The two sinkhole populations were selected for this analysis because they represent discrete populations with every adult individual known and tagged. No intermediate populations between these two sites have been found in extensive surveys of surrounding forest habitat. This allows for extensive, near-total sampling for this assay. These two in situ populations, here called Sinkhole 1 (SH1) and Sinkhole 2 (SH2), were compared to living collections developed from seeds collected during fieldwork in 2010. The ex situ plants are curated as separate accessions, defined as collections derived from single, separate mother plants (three accessions from SH1, four accessions from SH2; see ZD Table 1).

ZD Table 1. Sampling Structure for *Zamia decumbens* Populations Used in This Study.

| Code | Source | Type | N Plants |
| --- | --- | --- | --- |
| SH1 | Sinkhole 1 | In situ | 195 |
| SH-101 | Accession 101 | Ex situ | 46 |
| SH-103 | Accession 103 | Ex situ | 34 |
| SH-108 | Accession 108 | Ex situ | 14 |
| SH2 | Sinkhole 2 | In situ | 180 |
| SH2-027 | Accession 27 | Ex situ | 21 |
| SH-031 | Accession 31 | Ex situ | 31 |
| SH2-085 | Accession 85 | Ex situ | 31 |
| SH2-135 | Accession 135 | Ex situ | 28 |

***Zamia lucayana***

*Zamia lucayana* (Bay Rush) is endemic to a single Caribbean island (Long Island, The Bahamas), where it is restricted to a narrow strip of coastal sand dunes, and is now considered critically endangered due to small population size, limited extent of occurrence, and pressure from residential development and sand mining (Calonje et al. 2013). Conservation genetic analysis indicates that *Z. lucayana* has significant genetic structure as seen via analysis of molecular variance, but is considered a single population for management purposes (Calonje et al. 2013). Bay rush has an extremely small range, with a single population restricted to a short, narrow band of coastal, limestone sand dunes, in a single population of ca. 1000 plants. Caribbean zamias (from Florida and the West Indies), such as Bay Rush, reach reproductive maturity faster and produce reproductive structures more often than rainforest zamias such as Sinkhole Cycad (Griffith et al. 2012, Clugston et al. 2018). Thorough sampling of leaflets of in situ individuals from throughout the native populations was performed, as detailed in Calonje et al. (2013) and summarized here and in ZL Table 1. Leaflet samples from dispersed individuals covering the geographic span of the subpopulations were collected and labelled, with an emphasis on even spatial distribution. This study compares the in situ Bay Rush samples to cultivated plants in MBC ex situ collections derived from the in situ population. The ex situ collection is seed-grown from in situ female plants. Within the single population, Bay Rush has three major sub-populations, termed Buckley’s, Hamilton’s and Petty’s (Calonje et al. 2013). We compared these three in situ sub-populations to living collections developed from seeds collected during fieldwork in 2009. The ex situ plants are curated as separate accessions, defined as collections derived from single, separate mother plants (7 accessions from Buckley’s, 5 accessions from Hamilton’s, and 4 accessions from Petty’s; see below).

ZL Table 1. Sampling structure for bay rush (*Zamia lucayana*) populations used in the current study

| **Name** | **Source** | **Type** | **N plants** |
| --- | --- | --- | --- |
| Plants from Buckley’s sub-population: 43 in situ, 101 ex situ | | | |
| Buckley’s | Wild | In situ | 43 |
| Accession 12 | 20090812b | Ex situ | 16 |
| Accession 13 | 20090813 | Ex situ | 12 |
| Accession 14 | 20090814 | Ex situ | 19 |
| Accession 15 | 20090815 | Ex situ | 8 |
| Accession 16 | 20090816 | Ex situ | 18 |
| Accession 17 | 20090817 | Ex situ | 14 |
| Accession 18 | 20090818 | Ex situ | 14 |

| Plants from Hamilton’s sub-population: 45 in situ, 77 ex situ | | | |
| --- | --- | --- | --- |
| Hamilton’s | Wild | In situ | 45 |
| Accession 11 | 20090811 | Ex situ | 11 |
| Accession 24 | 20090824 | Ex situ | 19 |
| Accession 25 | 20090825 | Ex situ | 17 |
| Accession 26 | 20090826 | Ex situ | 18 |
| Accession 27 | 20090827 | Ex situ | 12 |
| Plants from Petty’s sub-population: 33 in situ, 66 ex situ | | | |
| Petty’s | Wild | In situ | 33 |
| Accession 19 | 20090819 | Ex situ | 9 |
| Accession 20 | 20090820 | Ex situ | 14 |
| Accession 21 | 20090821 | Ex situ | 15 |
| Accession 22 | 20090822 | Ex situ | 28 |

**Species genotyping**

***Quercus boyntonii***

*Quercus boyntonii* DNA was extracted using Omega kits and a FastPrep-24 DNA homogenizer (MP Biomedical). After extraction, DNA was diluted to ~5ng/uL using elution buffer or HPCL H2O. The PCR reactions were carried out in a total volume of 10 μL, containing 2 μL of DNA template (5ng/μL) and 8 μL of master mix consisting of 1 x reaction buffer, 0.5 mM total dNTPs, 1.5 mM MgCl_2_, varying amounts of each primer (see appendix), 0.5 μg/μL BSA, 0.025 U of GoTaq G2 DNA polymerase (Promega, Madison, Wisconsin, USA), and HPLC H2O to bring the reaction total to volume. Cycling conditions were: an initial denaturation step at 94°C for 5 min, then 30 cycles consisting of: 30 s at 94°C, 30 s at 50-56°C (Multiplex 1 @ 50°C, multiplex 2 @ 54°C, and multiplex 3 @ 56°C, Table 2) and 30 s at 72°C, followed by a final extension of 5 min at 72°C. PCRs were conducted on Eppendorf Mastercycler pro (Eppendorf, Hamburg, Germany) and C-1000 Touch (Bio-Rad, Hercules, California, USA) machines. A mix of 0.75 μL of each PCR product, 0.25 μL 600 LIZ size standard v2.0 (Applied Biosystems), and 9 μL Hi-Di™ Formamide (Applied Biosystems, USA), for a total of 10μL, was analyzed on an ABI 3730 Genetic Analyzer (Applied Biosystems).

Individuals were genotyped at 15 microsatellite markers (QB Table 3) that were found to have clear, consistent bands when visualized using gel electrophoresis. Forward primers were labeled with one of the following fluorescent dyes: 6-FAM, NED, VIC, or PET (Applied Biosystems, Waltham, Massachusetts, USA). Because genetic markers have not been optimized for this species, and we used markers that were previously used in *Quercus*, several were dropped due to issues such as “stutter” peaks, poor amplification, or frequently exhibiting more than two alleles. After identifying markers with consistent amplification and variability, we selected 11 markers for final analysis (QB Table 4). Microsatellite peaks were visualized and scored using Geneious software. We used Microchecker (Van Oosterhaut et al. 2004) to check for null alleles. We used the R package “poppr” to detect clones and removed these from our dataset before proceeding.

QB Table 3: Multiplex and Locus Summary

| **Multiplex** | **Locus** | **Dye** | **Complexity*** | **Published Repeat Unit** | **Previously Published Size Range (bp)** | **Realized Size Range (bp)** | **Kept for final analysis?** |
| --- | --- | --- | --- | --- | --- | --- | --- |
| MP1 | QS03797 | 6-FAM | Di | (CA)7 | 150-175 | - | No |
|  | MSQ13 | 6-FAM | Di | (TC)14 | 225-275 | - | No |
|  | QrZAG20 | VIC | Di | (TC)18 | 175-250 | 154.6-185.1 | Yes |
|  | QpZAG110 | NED | Di | (AG)15 | 175-225 | 199.6-230.4 | Yes |
|  | QS00314 | PET | Tri | (GAA) 6 | 175-200 | 166.6-210.4 | Yes |
|  | QpZAG9 | PET | Di | (AG)12 | 250-300 | 230.4-282.6 | Yes |
| MP2 | QS1904 | 6-FAM | Di | (TC) 10 | 125-175 | 134.9-159.8 | Yes |
|  | QS03297 | 6-FAM | Di | (CA)7 | 225-300 | 206.1-245.7 | Yes |
|  | MSQ4 | VIC | Di | (AG)17 | 200-300 | 194.8-257.5 | Yes |
|  | QS01386 | PET | Di | (CT)6 | - | - | No |
| MP3 | QS00562 | 6-FAM | Di | (GA) 7 | - | 188.3-227.3 | Yes |
|  | QpZAG119 | VIC | Di | (GA)24 | 64-98 | - | No |
|  | QrZAG87 | NED | Di | (TC)20 | 110-131 | 93.2-99.5 | Yes |
|  | QpZAG1/5 | PET | Complex | (GT)5(GA)9 | 160-190 | - | No |
|  | QM69-2M1 | PET | Complex | (TGG)6(CGG)(TGG)2 | 217 | 220.1-267.6 | Yes |

**Di = dinucleotide repeat: Tri=trinucleotide repeat; complex= complex repeat*

QB Table 4: List of primers sequences and source kept for final analysis.

| **Locus** | **5’ Primer Sequence** | **3’ Primer Sequence** | **Source** |
| --- | --- | --- | --- |
| QrZAG20 | CCA TTA AAA GAA GCA GTA TTT TGT | GCA ACA CTC AGC CTA TAT CTA GAA | Kampfer et al. 1998 |
| QpZAG110 | GGA GGC TTC CTT CAA CCT ACT | GAT CTC TTG TGT GCT GTA TTT | Steinkellner et al. 1997 |
| QS00314 | TCA AAA CGC AAC GTT TCA AG | TTC GGG TTT TCT TTG TGG TC | Chatwin et al. 2014 |
| QpZAG9 | GCA ATT ACA GGC TAG GCT GG | GTC TGG ACC TAG CCC TCA TG | Steinkellner et al. 1997 |
| QS1904 | TCA GTC AAA AAC CCA CCT CC | GGG TTT TCT TCA GTT TGC TTG T | Chatwin et al. 2014 |
| QS03297 | ACA CAA AGA GCC ATT CGC TT | GAG GCA TAC CTA CGG GAC AA | Chatwin et al. 2014 |
| MSQ4 | TCT CCT CTC CCA TAA ACA GG | GTT CCT CTA TCC AAT CAG TAG TGA G | Dow et. al. 1995 |
| QS00562 | ACC CCC ACC TAA TCC CAA C | TGC AAA CAC ACA GAG ACA CTT TT | Chatwin et al. 2014 |
| QrZAG87 | TCC CAC CAC TTT GGT CTC TCA | GTT GTC AGC AGT GGG ATG GGT A | Kampfer et al. 1998 |
| QM69-2M1 | CAC AAT CTG CCC ACA TCA TC | GGA TGG ACG AAG AGA AAG AT | Isagi &Suhandono, 1997 |

***Quercus georgiana***

Genomic DNA was extracted from fresh leaf tissue stored at -80°C using Qiagen miniprep extraction protocol.

Two types of DNA markers were examined. First polymorphic nuclear microsatellite markers were identified based on their successful use in *Q. rubra* L. and other red-oak group species (QG Table 3) (Aldrich et al. 2002, Aldrich et al. 2003). DNA was amplified using a polymerase chain reaction (PCR) using M13-labelled forward primers. Genotypes were scored using a CEQ 8000 Genetic Analysis System and CEQ Fragment Analysis software (Beckman Coulter).

QG Table 3: Microsatellite loci utilized

| **Locus** | **Repeat** | **Primer Sequence (5’-3’)** | **Size range (bp)** |
| --- | --- | --- | --- |
| QU.i21 | (GA)_16_ | F: ATATGGTCCCGATTAATTC  R: GGCAACATTCAAATGTATCTA | 189-219 |
| QU.M07 | (GA)_19_ | F: TTTAGCATCACATTTCCGTT  R: TTTTGTGTCATCCGGTATTA | 201-229 |
| QU.F02 | (GA)_15_ | F: CCAATCCACCCTTCCAAGTTCC  R: TGGTTGTTTTGCTTTATTCAGCC | 165-205 |
| QU.C19 | (GA)_18_ | F: TTAGCTTTTACGCAGTGTCG  R: CGGCTTCGGTTTCGTC | 234-264 |
| QU.E09 | (GA)_16_ | F: TGCCATCCCTATACACAACCA  G: CCTCCATCACAAAGTTGCC | 192-232 |
| QU.C08 | (GA)_29_ | F: TCCCAATCGATGTTTGATAAGG  R: GGGCTCTTGAGAGGATGTAGG | 271-309 |
| QU.G07 | (GA)_23_ | F: GCCAACAAATTTAACTATCCAT  R: TAACTGGGCTAGATAATCAG | 224-244 |
| QU.H14 | (GA)_22_ | F: GCTTGGGCTTGTTCCTACT  R: CAACACTTCTCATGGATTAGAGA | 279-325 |

Second, genic microsatellites (Expressed Sequence Tag- Simple Sequence Repeats, EST-SSRs) were originally developed and genetically mapped in *Quercus robur* (Durand et al. 2010, Bodénès et al. 2012). All markers listed in QG Tabl 4 has been adapted for use in the North American oak species (section Lobatae) *Quercus rubra* and *Quercus ellipsoidalis* (Sullivan et al. 2013, Lind-Riehl et al. 2014). Functional annotations were assigned to EST-SSRs using the Blast2GO software (Conesa et al. 2005). A total of 27 EST-SSRs have been tested for locus-specific amplification in eight *Q. georgiana* samples from 4 populations, out of which 14 have been selected for the analysis of all samples.

QG Table 4**.** Description of genic EST-SSRs

| **Locus** | **Repeat motif** | **Forward primer sequence (5’-3’)** | **Reverse primer sequence (5’-3’)** | T_a_ (C°) | **Size range (in base pairs)** |
| --- | --- | --- | --- | --- | --- |
| FIR013 | (CAG)5 | 6-FAM CGGGGAGGTTGATGAGTATT | AACACTGTCACCCCCATAGC | 56 | 133-144 |
| FIR039 | (CT)7 | PET-GAGCCTCTTTCATCGCTCAC | TCAACACCCCAAAACTCCAT | 59 | 111-132 |
| FIR043 | (TC)9 | PET-TTCTCCATTTCACACGCTTC | ACGACATCGTTTTGGAGCTT | 56 | 114-146 |
| FIR048 | (CT)9 | PET-TGCACCAAAATTGGAGGATG | TTGATGCAAGGTGCAGTTTC | 56 | 187-219 |
| GOT037 | (CT)11 | PET-CCATCCTTTTCATTCTTTCCA | TGTTGTTGTTGCTGTTGTCG | 57 | 239-265 |
| PIE039 | (CTT)8 | 6-FAM GTAAAACGACGGCCAGTGTCCTCACCCTCTGCGGTCT | CAGAAAGGGCTGCAAAGC | 59 | 157-178 |
| PIE200 | (CAA)5 | 6-FAM ACAACATGTGCCAAAACTGC | TCGATGATGTGGTTGTTGATG | 56 | 107-119 |
| PIE125 | (GGAAGC)3 | PET-AATACAAATCGCAGGAGGTG | CTAACCCATCGTTCATGGAG | 57 | 146-162 |
| FIR035 | (AT)6 | NED-GCTAAGGTTCCGTGTTCCAA | GGCCAGCAACTAAACCAAGA | 56 | 146-152 |
| FIR028 | (TC)8 | VIC-GGAAGAGTGTTCGGAAAGCA | CCAGCTCCTCCACAATAGCA | 56 | 201-237 |
| VIT081 | (CAT)3 | 6-FAM AATTCAAACCCAGCCAACTG | TCCTCTGGATGCTCCATCA | 56 | 112-136 |
| VIT086 | (CAG)5 | VIC-AAGAACACCCATTTCCACCA | TAAAATCCATTTGCCGGTTC | 56 | 184-207 |

PCR amplification followed the protocol described in Lind and Gailing (2013). Specific annealing temperatures are listed in Table 1. Specifically, samples were amplified in a GeneAmp PCR system 2700 (Applied Biosystem) with the following program: initial denaturation at 95 °C for 15 min followed by 35 cycles of denaturation at 94 °C for 45 sec, annealing at T_a_ (Table 1) for 45 sec and extension at 72 °C for 45 sec. The final extension step was for 20 min at 72 °C. Each PCR was executed in a 15 μl reaction mix composed of 5 μl HotFIREPol (Oak Biotechnologies, containing 10 mM MgCl_2_, 0.6 units of HOT FIREPol® Taq polymerase, and 2 mM of each dNTP;), 2 μl fluorescently labeled forward primers (5 μM), 2 μl reverse primers (5 μM), 2 μl DNA (~2 ng), and 4 μl molecular grade ultra pure water (Phenix Research Labs). PCR amplicons were separated on an ABI 3730 Genetic Analyzer with the internal size standard GS-LIZ-500 and scored with GeneMapper v. 4.0 (Applied Biosystems).

***Quercus oglethorpensis***

A minimum of 1 cm^2^ of leaf tissue was collected from each *Q. oglethorpensis* individual. Leaf samples were dried in silica gel and stored at -20ºC for later DNA extraction. We extracted total genomic DNA from silica-dried leaf tissue following a CTAB method modified from Doyle and Doyle (1987). DNA samples were purified to a final elution volume of 30–50 μl using the QIAquick PCR Purification Kit (QIAGEN, Germany). Nuclear microsatellite primers were selected for screening based on prior successful use in *Quercus* subgenus *Quercus* (sections *Quercus* and *Lobatae*) and subgenus *Cyclobalanopsis* (Chatwin et al., 2014; Dow and Ashley, 1996; Isagi and Suhandono 1997; Kampfer et al. 1998; Steinkellner et al. 1997). We screened a total of 42 total markers using 15 DNA samples. 38 markers amplified, of which 21 were polymorphic and 17 were monomorphic. Genotypes were obtained for 6 reliably polymorphic markers: QP(zag)9, QS009, QP(zag)16, QR(zag)15, QR(zag)30. To visualize alleles, primer pairs were labeled with either an M13 sequence to the 5' end and then labeled with WellRED (Sigma-Adrich, Germany) cyanine-based fluorescent dye at the 5' end [QP(zag)9, QS009, QP(zag)16] or primer pairs were designed with the WellRED fluorescent dye at the 5' end [QR(zag)15, QR(zag)30, MAQ4, QS017, ZAG20, QP110, QpZAG1-2, ZAG111, QS003, QP110f, QpZag1/5].

For M13 labeled primer pairs the initial PCR reaction was performed in a 10µL reaction containing 2-15 ng of DNA, 0.2 µL of forward and reverse primer, 3.5 µL of DNA grade water, and 5µL of MyTaq™ master mix (Bioline, United States). PCR reactions were performed in a Mastercycle pro® thermocycler (eppendorf, Germany) under the following conditions: 2 minutes initial annealing at 94ºC, then 25 cycles at 94ºC for 30 seconds, 55ºC for 30 seconds, and 72ºC for 45 seconds, and a final extension at 72ºC for 5 minutes. The initial PCR was product was then labeled in a second PCR reaction performed with the addition of 2.5µL of MyTaq™ master mix 2.1 µL of DNA grade water, and M13 primer labeled with labeled with WellRED cyanine-based fluorescent dye D3 or D4. For primer pairs designed with WellRED dyes the PCR reaction was performed in a 10 µL reaction containing 2-15 ng of DNA, 0.2 µL of forward and reverse primer, 3.5 µL of DNA grade water, and 5 µL of MyTaq™ master mix. PCR reactions were performed in a Mastercycle pro® thermocycler under the following conditions: 2 minutes initial annealing at 94ºC, then 35 cycles at 94ºC for 30 seconds, 55ºC for 30 seconds, and 72ºC for 45 seconds, and a final extension at 72ºC for 5 minutes. Final PCR products were multiplex and analyzed on a CEQ 8000 Genetic Analysis System and CEQ Fragment Analysis software (Beckman Coulter, United States) using GenomeLab400 internal size standard (ABSCIEX, United States).

QO Table 3. Summary of 6 microsatellite loci developed for species within *Quercus* section *Quercus* (white oak) that were used in this study of *Quercus oglethorpensis*.

| **Locus** | **Source** | **primer forward &**  **primer reverse (5’-3’)** | **Repeat Motif** | **Fluorescent Label** | **Size Range (bp)** |
| --- | --- | --- | --- | --- | --- |
| QPZAG16 | Steinkellner et al. 1997 | F: CTTCACTGGCTTTTCCTCCT  R: TGAAGCCCTTGTCAACATGC | (AG)n | M13  [D4-PA] | 159-189 |
| QPZAG9 | Steinkellner et al. 1997 | F: GCAATTACAGGCTAGGCTGG  R: GTCTGGACCTAGCCCTCATG | (AG)n | M13  [D3-PA] | 244 – 290 |
| QS00984 | Chatwin et al. 2014 | F: TTTCATTTCAAGAAACAACAAGTGA  R: CAATCTCATCGTCCAAGCCT | (AAT)n | M13  [D3-PA] | 156 - 168 |
| MSQ4 | Dow et. al. 1995 | F: TCTCCTCTCCCCATAAACAGG  R: GTTCCTCTATCCAATCAGTAGTGAG | (AG)n | [D3-PA] | 200 - 234 |
| QRZAG15 | Kampfer et al. 1998 | F: CCTCCTAACAGTAACATTCTACGA  R: GTGGTATCTATATCTTGCCAAAGG | (GA)n | [D3-PA] | 134 - 200 |
| QRZAG30 | Kampfer et al. 1998 | F: TGCTCCGTCATAATCTTGCTCTGA  R: GCAATCCTATCATGCACATGCACAT | (GA)n | [D4-PA] | 170 - 200 |

***Magnolia ashei***

*Magnolia ashei* DNA extraction and PCR are described in Von Kohn et al. (2018).

**Magnolia pyramidata**

*Magnolia pyramidata* DNA was extracted using Omega kits and a FastPrep-24 DNA homogenizer (MP Biomedical). After extraction, DNA was diluted to ~5ng/uL using elution buffer or HPCL H2O. The PCR reactions were carried out in a total volume of 12 μL, containing 2 μL of DNA template (5ng/μL) and 10 μL of master mix consisting of 1 x reaction buffer, 0.2 mM total dNTPs, 1.5 mM MgCl_2_, varying amounts of each primer (see appendix), 0.8 μg/μL BSA, 0.041 U of GoTaq G2 DNA polymerase (Promega, Madison, Wisconsin, USA), and HPLC H_2_O to bring the master mix to volume. Forward primers were labeled with one of the 6-FAM, NED, VIC, or PET fluorescent dyes (Applied Biosystems, Waltham, Massachusetts, USA, Table 3). Cycling conditions were: an initial denaturation step at 94°C for 5 min, then 20 cycles consisting of 30 s at 94°C, 30s at step down protocol starting at 60°C minus 0.2 °C/cycle, and 30 s at 72°C, followed by 20 cycles consisting of 30 s at 94°C, 30s at 50°C, and 30 s at 72°C, ending with a a final extension of 1 min at 72°C. PCRs were conducted on Eppendorf Mastercycler pro (Eppendorf, Hamburg, Germany). Amplification products were visualized using a 1.5% agarose, 0.5 x TAE gel with ethidium bromide stain and sized with a 1kb+ ladder (Thermofisher Scientific, Waltham, Massachusetts, USA). A mix of 0.75 μL of each PCR product, 0.25 μL 600 LIZ size standard v2.0 (Applied Biosystems), and 9 μL Hi-Di™ Formamide (Applied Biosystems, USA), for a total of 10μL, was analyzed on an ABI 3730 Genetic Analyzer (Applied Biosystems).

Individuals were genotyped at 18 microsatellite markers (Table 3) that were found to have clear, consistent bands when visualized using gel electrophoresis. Because genetic markers have not been optimized for this species, and we used markers that were previously used in *Magnolia*, as well as had a handful designed, several were dropped due to issues such as “stutter” peaks, poor amplification, or frequently exhibiting more than two alleles. After identifying markers with consistent amplification and variability, we selected 11 markers for final analysis (Table 4). Microsatellite peaks were visualized and scored using Geneious software. We used Microchecker (Van Oosterhaut et al. 2004) to check for null alleles. We used the R package “poppr” to detect clones and removed these from our dataset before proceeding.

MP Table 3: Multiplex and Locus Summary

| **Multiplex** | **Locus** | **Dye** | **Complexity** | **Repeat Unit** | **Expected Range (bp)** | **Realized Range (bp)** | **Kept for final analysis?** |
| --- | --- | --- | --- | --- | --- | --- | --- |
| MP1 | mpy85 | 6-FAM | Di | (ga)25 | 153-213 | 138-194 | Yes |
|  | mpy93 | VIC | Di | (gt)12 | 89-149 | 115-122 | Yes |
|  | mpy11 | VIC | Di | (gt)20 | 190-250 | -- | No |
|  | mpy83 | NED | Di | (tc)19 | 168-228 | 195-201 | Yes |
|  | mpy78(2) | PET | Di | ct(17) | 136-196 | 165-179 | Yes |
|  | mpy16 | PET | Di | (ga)13 | 237-297 | -- | No |
| MP2 | mpy81(2) | 6-FAM | Complex | CAGAGAGAGAGAGAGAGAGAGAGAGAGA | 145-205 | 163-210 | Yes |
|  | mpy15 | VIC | Di | (gt)28 | 87-147 | 83-134 | Yes |
|  | mpy17 | VIC | Complex | (ga)22-9-(ga)10 | 198-258 | 196-232 | Yes |
|  | mpy82 | NED | Di | (gt)12 | 128-188 | -- | No |
|  | mpy20 | NED | Di | (ga)19 | 229-289 | -- | No |
|  | mpy96b | VIC | Di | (ct)24 | 154-214 | 154-206 | Yes |
| MP3 | stm0214 | 6-FAM | Di | (GT)18 | 121-143 | -- | No |
|  | mpy72(2) | 6-FAM | Complex | TTTACACACGCACACACACCACCACA | 184-244 | 198-217 | Yes |
|  | M6D3 | VIC | Dii | (CT)22 | 101-161 | 111-158 | Yes |
|  | mpy80(2) | VIC | Complex | TGAGAGAGAGAGAGAGAGAGAGAGAG | 229-289 | -- | No |
|  | stm0200 | NED |  | (CT)13(TC)11 | 167-211 | -- | No |
|  | M6D8 | PET | Complex | (CT)3C(CT)10 | 140-200 | 144-211 | Yes |

*Di = dinucleotide repeat: Tri=trinucleotide repeat; complex= complex repeat

Table 4: List of primers sequences and source kept for final analysis.

| **Locus** | **5’ Primer Sequence** | **3’ Primer Sequence** | **Source** |
| --- | --- | --- | --- |
| mpy85 | GTTTCAAGGTGGGTGGAAAA | CCCATCACTCCATCACCTTT | Genetic Marker Services |
| mpy93 | gtacCCATAGGTGGTGAAAT | ATTATGGTATCCGTAATTTGTGTG | Genetic Marker Services |
| mpy83 | CGGGAAAGTGAGTGGAAGAG | AAGCCTATTTTGGGCATGAA | Genetic Marker Services |
| mpy78(2) | ACT CAC CTT GAT GAA TTG GA | TCC ATA AAT ACC CCT CCA TC | Genetic Marker Services |
| mpy81(2) | AAC ATG GAA GTA AGC GAC AT | gtacGACCACGTTGTAA | Genetic Marker Services |
| mpy15 | TTC TAT TGC TCT CTA GAT GGA TGC | ACC AAC AAG TCA ACA ACA AAA A | Genetic Marker Services |
| mpy17 | GTC GGA GCA CCG TTA CAA TG | TAC CAC GCA CAT GGC TGA TT | Genetic Marker Services |
| mpy96b | TGGTGGGACCCATATTGATT | TACGGCATGGATCAAGTGGA | Genetic Marker Services |
| mpy72(2) | AAT AAA ATG TGT AGC CTT TGG A | TCA GAT TAA GCT GTC CCA AC | Genetic Marker Services |
| M6D3 | ACA TGG ATA GTC GTT GGA TA | ACC CCA CTG AAG ACA AAC AT | ISAGI et al. 1999 |
| M6D8 | CGA GTG GCA TTT CCG TAA TA | GAA CCT GGC GCA CCG TAG TC | ISAGI et al. 1999 |

***Hibiscus waimeae* subsp. *hannerae* and *H. waimeae* subsp. *waimeae***

Genomic DNA was extracted using the modified 2× CTAB method (Doyle and Doyle*,* 1987). We initially tested over 41 genetic markers developed for a number of members of *Hibiscus* species (11 from *H. aridicola* (Zhang et al. 2011), 8 from *H. glaber* (Ohtani et al. 2008), 10 from *H. rosa-sinensis* (Bruna et al. 2009), and 12 from *H. tiliaceus* (Takayama et. al 2006)). Of these only eight amplified in *Hibiscus waimeae* subspecies and only three proved to be polymorphic (HA-13, H-DAT1, H-MALP4). Due to low number of working primers, we sent genomic DNA to the microsatellite-development company, Genetic Marker Services (Brighton, UK; [www.geneticmarkerservices.com](http://www.geneticmarkerservices.com/)) to develop new primer pairs. Libraries were constructed using adaptor-ligation of digested genomic DNA, which was then screened with filter-bonded synthetic repeat motifs: [AG]17, [AC]17, [AAC]10, [CCG]10, [CTG]10, and [AAT]10. These were transformed into *E. coli*, plated onto agar/ampicillin plates and screened for motif-positive clones which were then isolated, cultured and sequenced. To help minimize later multi-loading overlap ambiguities during sequencer genotyping primers were designed using the online primer design software PRIMER 3.0 (Rozen and Skaletsky, 2000). A total of 16 new primer pairs were designed that amplified products ranging from 100–300 bp, of these only five were polymorphic (hwa56, hwa71, hwa72, hwa73, hwa75). To visualize and quantify allele sizes, the forward primer derived from *B. insignis* libraries was modified with WellRed Black (D2), Green (D3) or Blue (D4) fluorescent dye (Sigma-Proligo, St. Louis, Missouri, USA). The PCR was set up using 10-50ng template DNA, 25 μM of modified forward and reverse primer and Bioline PCR MasterMix 2x (Bioline, Tauton, Massachusetts) and run at 94^o^C for 3 mins, followed by 35 cycles of 94^o^C for 40 s, 57^o^C for 40 s, and 72^o^C for 90 s, with a final extension of 72^o^C for 10 min. All products were analyzed and scored using a CEQ 8000 Genetic Analysis System V9.0 (Beckman Coulter, Brea, California, USA).

HW Table 4: Primer information

| **Primer Name** | **Repeat motif** | **Primer** | **Sequence (5’-3’)** | **Size range** | **No alleles** | **Species of origin** | **Source** |
| --- | --- | --- | --- | --- | --- | --- | --- |
| HA-13 | (CTT)15 | F | ACTTTTATCGTATAGACCAG | 110-118 | 6 | *Hibiscus aridicola* | Zhang et al. 2011 |
|  |  | R | GAACACCTTTATTTCAGTGT |  |  |  |  |
| HA-22 | (AC)9 | F | ACTGGTAACATCCCTGAC | 107 | 1 | *Hibiscus aridicola* | Zhang et al. 2011 |
|  |  | R | GAAACTGCTGGAAATCTA |  |  |  |  |
| HA-27 | (TG)10 | F | TGAATTTCTTTTCTTCCTTTAC | 207 | 1 | *Hibiscus aridicola* | Zhang et al. 2011 |
|  |  | R | CAACTATCATCTTGTCGTGC |  |  |  |  |
| HA-37 | (GT)11 | F | TAAGATGGTATTGGAAGGG | 345 | 1 | *Hibiscus aridicola* | Zhang et al. 2011 |
|  |  | R | AGGGAGCATAAAAGTGGT |  |  |  |  |
| H-DAT1 | (TGC)6 | F | CCCTTCAAGTGCTCCTCT | 134-164 | 7 | *Hibiscus rosa-sinensis* | Bruna et al. 2009 |
|  |  | R | TCAATTCACCTTCCGTACCC |  |  |  |  |
| H-DAT3 | (TA)20 | F | AAGCGAAATCGACTGAAGGA | 450 | 1 | *Hibiscus rosa-sinensis* | Bruna et al. 2009 |
|  |  | R | TGTCGTAGAAACTTCCAATCCA |  |  |  |  |
| H-MALP1 | (CT)8 | F | AGCCTGTCACCAACAAA | 159 | 1 | *Hibiscus rosa-sinensis* | Bruna et al. 2009 |
|  |  | R | GAGAGCTTACGAAGCGGAGA |  |  |  |  |
| H-MALP4 | (AC)2 (AT)4 | F | CACCNCAAACATACTCACAC | 221-265 | 20 | *Hibiscus rosa-sinensis* | Bruna et al. 2009 |
|  |  | R | ACTGTGCAGCCACTTCAACA |  |  |  |  |
| hwa56 | (GT)9 | F | CCA TGA ATG AGA GAC AGC AAA G | 105-113 | 5 | *Hibiscus waimeae* subsp. | Genetic Marker Services |
|  |  | R | TTG TCA AAA TTG GTC CAT ACC |  |  |  |  |
| hwa71 | (CT)12 | F | GAGGATGCGTGGTAGGTTGT | 170-194 | 7 | *Hibiscus waimeae* subsp. | Genetic Marker Services |
|  |  | R | CCGTGGACACCCGTAATACT |  |  |  |  |
| hwa72 | (GA)12 | F | TCACTCAACCAAAGCAGACG | 219-262 | 28 | *Hibiscus waimeae* subsp. | Genetic Marker Services |
|  |  | R | ACCTGGTCAGCTTCAGCAGT |  |  |  |  |
| hwa73 | (CT)16 | F | CGTTGGATAAAGAGTAATCCAAGAT | 172-231 | 23 | *Hibiscus waimeae* subsp. | Genetic Marker Services |
|  |  | R | CAACTGCTGTTCCGCCTATT |  |  |  |  |
| hwa75 | (CT)17 | F | GGTGAAATGAAAACCGAATCA | 159-175 | 24 | *Hibiscus waimeae* subsp. | Genetic Marker Services |
|  |  | R | ATGGGATTTCGATCCATTCA |  |  |  |  |

***Pseudophoenix ekmanii* and *P. sargentii***

DNA isolation, PCR amplification and visualization of SSR fragments follow protocols detailed by Meerow and Nakamura (2007). Total DNA was extracted from dried leaflets via FastDNA Kit (MP Biomedicals, LLC). We selected ten microsatellite primers (Namoff et al. 2010a) for this analysis: pse2.1, pse3.11, pse3.33b, pse3.34b, pse3.6, pse5.2 pse5.4, pse5.5, pse5.6, and pse7.26b. This primer set is well established as an appropriate assay for the genus *Pseudophoenix* (Rodríguez-Peña et al. 2014a, b). PCR mix was 1× buffer (15 mm MgCl2), 200 µm dNTPs, 250 nm each of forward and reverse primers, 0.25 U Taq DNA polymerase (New England Biolabs), 10 ng genomic DNA template, and nuclease‐free distilled water up to a total volume of 10 µL. The following PCR program was run on an ABI 9700 thermocycler (Applied Biosystems): 2 min at 94 °C, 38 cycles of (30 s at 94 °C, 1 min at 54–68 °C depending on primer per Namoff et al. (2010a), 1 min at 72 °C), 10 min at 72 °C, and storage at 4 °C. Allele size was detected on an ABI 3730 Genetic Analyser (Applied Biosystems) via capillary gel electrophoresis alongside a genescan ROX‐500 size standard (Applied Biosystems). Raw microsatellite data was analyzed with Genemapper 4.0 (Applied Biosystems).

***Zamia decumbens* and *Z. lucayana***

DNA isolation, PCR amplification, and subsequent visualization of simple sequence repeat fragments also follow the protocols described by Meerow and Nakamura (2007). Total DNA was extracted from dried leaflets via DNeasy-Plant-Mini-Kits (Qiagen). We used 10 DNA microsatellites for this analysis, which were developed for Caribbean Zamia studies: Zam28, Zam33, Zam53, Zam59, Zam60, Zam61, Zfg23, Zfg25, Zfg32, and Zfg33, following the protocols of Meerow et al. (2012). PCR mix was 1× buffer (15 mm MgCl2), 200 µm dNTPs, 250 nm each of forward and reverse primers, 0.25 U Taq DNA polymerase (New England Biolabs), 10 ng genomic DNA template, and nuclease‐free distilled water up to a total volume of 10 µL. PCR was performed on an ABI 9700 thermocycler (Applied Biosystems) using the following program: 2 min at 94 °C, 38 cycles of (30 s at 94 °C, 1 min at 54–68 °C depending on primer per Namoff et al. (2010a), 1 min at 72 °C), 10 min at 72 °C, and storage at 4 °C. We detected allele size using an ABI 3730 Genetic Analyser (Applied Biosystems) via capillary gel electrophoresis compared alongside a genescan ROX‐500 size standard (Applied Biosystems). Analysis of raw microsatellite data was performed using Genemapper 3.5 (Applied Biosystems).

**Additional results**

**Genetic diversity statistics for all wild populations for each species**

Each row is a population. All calculations were made using R packages adegenet and hierfstat. Only unique multilocus genotypes were used for analysis- i.e. for each clone pair identified one was removed. This table excludes populations less than 5 individuals (which is one population from each of: *Quercus oglethorpensis*, *Quercus georgiana*, *Quercus boyntonii* and *Pseudophoenix sargentii*) because very small populations will cause distortions in the allelic richness and F_ST_ calculations

|  | **Samples** | **unique multi locus genotypes** | **expected heterozygosity** | **number of alleles** | **allelic richness per locus** | **mean pairwise F_ST_** |
| --- | --- | --- | --- | --- | --- | --- |
| *Hibiscus* w. *hannerae* | 48 | 46 | 0.634 | 38 | 3.964 | 0.033 |
|  | 30 | 29 | 0.651 | 40 | 4.250 | 0.039 |
|  | 6 | 6 | 0.442 | 14 | 2.333 | 0.029 |
|  | 74 | 71 | 0.728 | 53 | 4.782 | 0.026 |
| *Hibiscus* w. *waimeae* | 30 | 30 | 0.536 | 41 | 2.354 | 0.061 |
|  | 13 | 13 | 0.555 | 32 | 2.327 | 0.06 |
|  | 31 | 31 | 0.507 | 35 | 2.206 | 0.071 |
| *Magnolia pyramidata* | 22 | 22 | 0.800 | 117 | 7.933 | 0.069 |
|  | 61 | 61 | 0.757 | 134 | 7.28 | 0.055 |
|  | 10 | 10 | 0.715 | 65 | 5.909 | 0.075 |
|  | 21 | 21 | 0.531 | 46 | 3.531 | 0.125 |
| *Magnolia ashei* | 57 | 55 | 0.482 | 63 | 3.445 | 0.171 |
|  | 48 | 48 | 0.316 | 49 | 2.714 | 0.171 |
| *Pseudophoenix ekmanii* | 99 | 98 | 0.429 | 53 | 5.249 | 0.028 |
|  | 103 | 102 | 0.39 | 45 | 4.439 | 0.028 |
| *Pseudophoenix sargentii* | 10 | 5 | 0.176 | 14 | 1.4 | 0.046 |
|  | 103 | 103 | 0.635 | 108 | 4.241 | 0.046 |
| *Quercus boyntonii* | 14 | 12 | 0.581 | 50 | 2.996 | 0.029 |
|  | 17 | 15 | 0.605 | 59 | 3.092 | 0.037 |
|  | 22 | 22 | 0.605 | 58 | 2.988 | 0.031 |
|  | 12 | 11 | 0.593 | 32 | 2.774 | 0.049 |
|  | 83 | 76 | 0.642 | 106 | 3.288 | 0.016 |
|  | 60 | 60 | 0.63 | 72 | 3.043 | 0.023 |
|  | 30 | 30 | 0.651 | 79 | 3.361 | 0.029 |
|  | 5 | 5 | 0.459 | 28 | 2.448 | 0.055 |
| *Quercus georgiana* | 24 | 24 | 0.62 | 115 | 5.593 | 0.054 |
|  | 25 | 25 | 0.572 | 121 | 5.781 | 0.051 |
|  | 26 | 26 | 0.612 | 112 | 5.405 | 0.063 |
|  | 24 | 24 | 0.625 | 111 | 5.385 | 0.047 |
|  | 25 | 25 | 0.584 | 116 | 5.643 | 0.046 |
|  | 24 | 24 | 0.576 | 98 | 4.833 | 0.051 |
|  | 26 | 26 | 0.624 | 127 | 5.992 | 0.042 |
|  | 25 | 25 | 0.686 | 143 | 6.831 | 0.039 |
|  | 25 | 25 | 0.637 | 124 | 6.064 | 0.042 |
| *Quercus oglethorpensis* | 26 | 26 | 0.588 | 57 | 5.033 | 0.074 |
|  | 14 | 14 | 0.645 | 59 | 5.9 | 0.069 |
|  | 33 | 33 | 0.694 | 76 | 6.026 | 0.052 |
|  | 27 | 27 | 0.639 | 73 | 6.252 | 0.054 |
|  | 31 | 31 | 0.653 | 68 | 5.556 | 0.073 |
|  | 29 | 29 | 0.62 | 70 | 5.747 | 0.059 |
|  | 28 | 28 | 0.648 | 74 | 6.058 | 0.055 |
| *Zamia lucayana* | 45 | 45 | 0.719 | 86 | 8.113 | 0.033 |
|  | 43 | 43 | 0.756 | 81 | 7.782 | 0.031 |
|  | 33 | 33 | 0.752 | 79 | 7.869 | 0.03 |
| *Zamia decumbens* | 195 | 177 | 0.574 | 40 | 3.992 | 0.127 |
|  | 180 | 173 | 0.618 | 70 | 6.989 | 0.127 |
|  | | | | | | |
|  | | | | | | |

**Allele capture curves and minimum sampling for 70% sufficiency**

Figure S1: Minimum sampling needed to capture 70% of the alleles for the Reduced Dataset


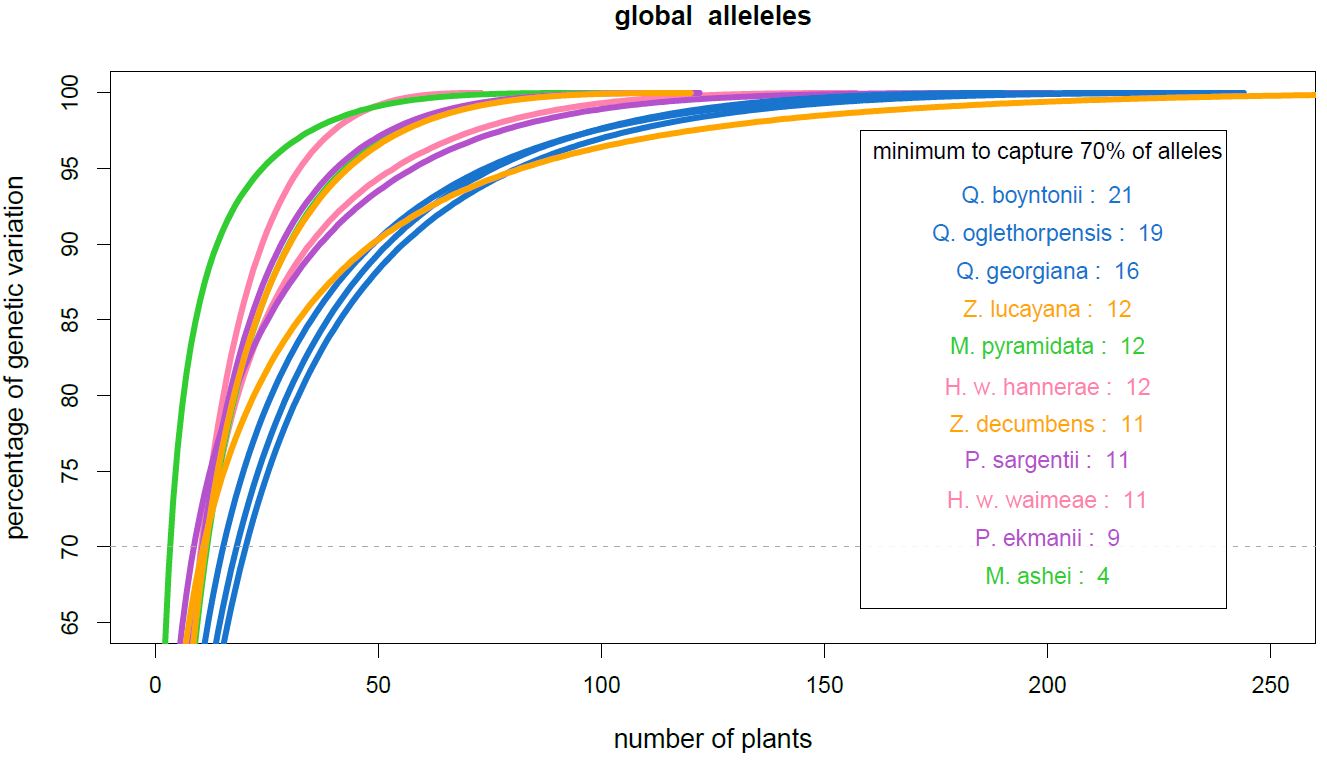


Figure S1: Minimum sampling needed to capture 70% of the alleles for the Full Dataset
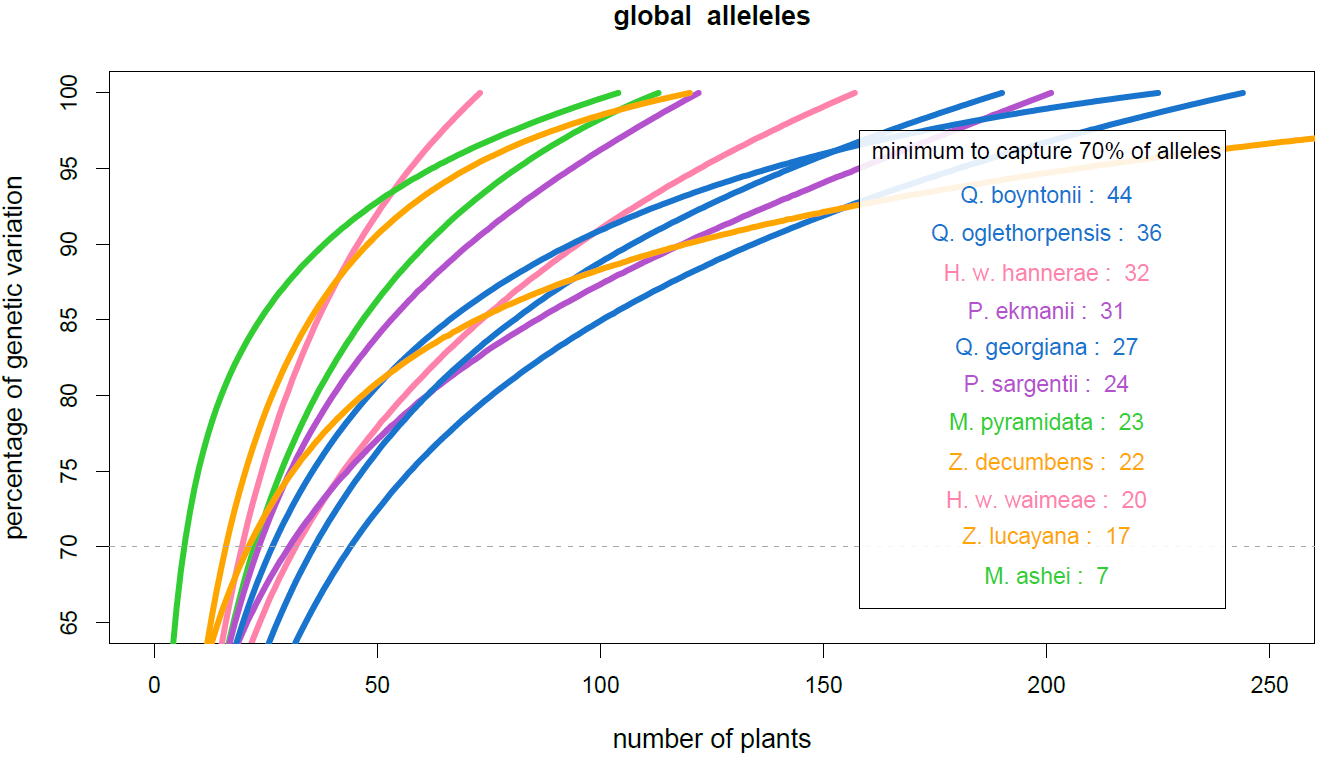


**P values from Statistical tests**

As described in the Methods, we tested whether F_ST_ among populations could predict the current proportion of genetic diversity conserved and/or the number of samples that are needed for sufficient sampling (e.g. 95% of the alleles). The first table below shows four summary statistics of the pairwise population F_ST_ values of each species. The second and third table shows the p values. Values shown are for the Reduced Dataset but values are very similar for Full Dataset, and can be calculated using provided code.

Table S1: Summaries of pairwise population FSTs for each species (calculated on the pairwise FST matrix)

|  | Mean | SD | Max | Min |
| --- | --- | --- | --- | --- |
| Hhannerae | 0.033 | 0.010 | 0.048 | 0.017 |
| Hwaimeae | 0.070 | 0.025 | 0.100 | 0.033 |
| Masheii | 0.090 | 0.063 | 0.171 | 0.042 |
| Mpyramidata | 0.065 | 0.043 | 0.147 | 0.027 |
| Pekmanii | 0.023 | 0.004 | 0.028 | 0.020 |
| Psargentii | 0.137 | 0.178 | 0.515 | 0.008 |
| Qboyntonii | 0.030 | 0.026 | 0.155 | 0.004 |
| Qgeorgiana | 0.043 | 0.013 | 0.080 | 0.018 |
| Qoglethorpensis | 0.050 | 0.024 | 0.093 | 0.008 |
| Zdecumbens | 0.071 | 0.045 | 0.129 | 0.041 |
| Zlucayana | 0.019 | 0.013 | 0.034 | 0.006 |

Table S2: P values of regressions of summaries of pairwise F_ST_ on the amount of genetic diversity of each type of allele captured (e.g. the percentage value from the main text Table 2). These are all raw (uncorrected) p values (the function p.adjust in R was used to correct for multiple comparisons)

|  | Mean | SD | Max | Min |
| --- | --- | --- | --- | --- |
| All | 0.434 | 0.366 | 0.384 | 0.785 |
| Common | 0.829 | 0.67 | 0.605 | 0.914 |
| Low Freq | 0.857 | 0.357 | 0.508 | 0.578 |
| Rare | 0.9 | 0.9 | 0.931 | 0.788 |

Table S3: P values of regressions of summaries of pairwise F_ST_ on the minimum sample size needed for each type of allele (e.g. the values shown in Figure 2). These are all raw (uncorrected) p values (the function p.adjust in R was used to correct for multiple comparisons)

|  | Mean | SD | Max | Min |
| --- | --- | --- | --- | --- |
| global thresh 70 | 0.14 | 0.998 | 0.782 | 0.018 |
| common thresh 70 | 0.584 | 0.239 | 0.55 | 0.03 |
| low freq thresh 70 | 0.349 | 0.551 | 0.617 | 0.46 |
| rare thresh 70 | 0.186 | 0.675 | 0.007 | 0.311 |
| global thresh 95 | 0.25 | 0.534 | 0.537 | 0.298 |
| common thresh 95 | 0.421 | 0.269 | 0.653 | 0.011 |
| low freq thresh 95 | 0.134 | 0.448 | 0.426 | 0.196 |
| rare thresh 95 | 0.047 | 0.866 | 0.055 | 0.084 |

As described in the Methods, we tested whether the allele frequency spectrum (specifically proportion of alleles below given thresholds) could predict the current proportion of genetic diversity conserved and/or the number of samples that are needed for sufficient sampling (e.g. 95% of the alleles). The first table below shows four summary statistics of the allele frequency spectra of each species. The second and third tables show the p values. Values shown are for the Reduced Dataset but values are very similar for Full Dataset, and can be calculated using provided code.

Table S3: The proportion of alleles that fall below thresholds (columns) of allele frequency, for each species (rows).

|  | **alleles with a.f.<0.5%** | **alleles with a.f.<1%** | **alleles with a.f.<5%** | **alleles with a.f<10%** |
| --- | --- | --- | --- | --- |
| **Hwaimeae** | 0.000 | 0.190 | 0.621 | 0.828 |
| **Hhannerae** | 0.260 | 0.390 | 0.623 | 0.818 |
| **Mashei** | 0.145 | 0.197 | 0.342 | 0.421 |
| **Mpyramidata** | 0.178 | 0.305 | 0.644 | 0.810 |
| **Pekmanii** | 0.306 | 0.339 | 0.548 | 0.661 |
| **Psargentii** | 0.254 | 0.328 | 0.623 | 0.770 |
| **Qgeorgiana** | 0.171 | 0.278 | 0.588 | 0.750 |
| **Qoglethorpensis** | 0.390 | 0.475 | 0.787 | 0.908 |
| **Qboyntonii** | 0.276 | 0.328 | 0.731 | 0.858 |
| **Zdecumbens** | 0.171 | 0.250 | 0.513 | 0.566 |
| **Zlucayana** | 0.121 | 0.177 | 0.581 | 0.758 |

Table S2: P values of regressions of summaries of the allele frequency spectrum on the amount of genetic diversity of each type of allele captured (e.g. the percentage value from the main text Table 2). These are all raw (uncorrected) p values (the function p.adjust in R was used to correct for multiple comparisons)

|  | **alleles with a.f.<0.5%** | **alleles with a.f.<1%** | **alleles with a.f.<5%** | **alleles with a.f<10%** |
| --- | --- | --- | --- | --- |
| All | 0.086 | 0.464 | 0.744 | 0.475 |
| Common | 0.012 | 0.195 | 0.921 | 0.665 |
| Low Freq | 0.069 | 0.23 | 0.471 | 0.808 |
| Rare | 0.944 | 0.982 | 0.839 | 0.946 |

Table S3: P values of regressions of summaries of the allele frequency spectrum on the minimum sample size needed for each type of allele (e.g. the values shown in Figure 2). These are all raw (uncorrected) p values (the function p.adjust in R was used to correct for multiple copmarisons)

|  | **alleles with a.f.<0.5%** | **alleles with a.f.<1%** | **alleles with a.f.<5%** | **alleles with a.f<10%** |
| --- | --- | --- | --- | --- |
| global thresh 70 | 0.417 | 0.161 | 0.001 | 0.013 |
| common thresh 70 | 0.901 | 0.307 | 0.002 | 0 |
| low freq thresh 70 | 0.13 | 0.247 | 0.282 | 0.776 |
| rare thresh 70 | 0.513 | 0.175 | 0.829 | 0.649 |
| global thresh 95 | 0.157 | 0.147 | 0.164 | 0.498 |
| common thresh 95 | 0.912 | 0.258 | 0.002 | 0 |
| low freq thresh 95 | 0.031 | 0.079 | 0.191 | 0.555 |
| rare thresh 95 | 0.312 | 0.062 | 0.69 | 0.295 |
